# Complex Living Conditions Impair Behavioral Inhibition but Improve Attention in Rats

**DOI:** 10.3389/fnbeh.2015.00357

**Published:** 2015-12-24

**Authors:** Rixt van der Veen, Jiska Kentrop, Liza van der Tas, Manila Loi, Marinus H. van IJzendoorn, Marian J. Bakermans-Kranenburg, Marian Joëls

**Affiliations:** ^1^Department of Translational Neuroscience, Brain Center Rudolf Magnus, University Medical Center UtrechtUtrecht, Netherlands; ^2^Centre for Child and Family Studies, Leiden UniversityLeiden, Netherlands

**Keywords:** enriched environment, adolescence, maternal deprivation, early life, behavioral inhibition, attention, 5-choice SRTT

## Abstract

Rapid adaptation to changes, while maintaining a certain level of behavioral inhibition is an important feature in every day functioning. How environmental context and challenges in life can impact on the development of this quality is still unknown. In the present study, we examined the effect of a complex rearing environment during adolescence on attention and behavioral inhibition in adult male rats. We also tested whether these effects were affected by an adverse early life challenge, maternal deprivation (MD). We found that animals that were raised in large, two floor Marlau^TM^ cages, together with 10 conspecifics, showed improved attention, but impaired behavioral inhibition in the 5-choice serial reaction time task. The early life challenge of 24 h MD on postnatal day 3 led to a decline in bodyweight during adolescence, but did not by itself influence responses in the 5-choice task in adulthood, nor did it moderate the effects of complex housing. Our data suggest that a complex rearing environment leads to a faster adaptation to changes in the environment, but at the cost of lower behavioral inhibition.

## Introduction

Today’s living environment has become drastically complex and demanding, with rapidly changing technologies asking for constant attention and quick adaptation. This also impacts on the development of children and their brains, shaping response patterns for life. Adaptation to the environment is, however, not always a guarantee to thrive in society. When prompt responding develops into more impulsive behavior, children might be at risk for worse outcomes later in life in terms of cognitive development, socio-economic status, and criminal offenses (Moffitt et al., 2011; Fergusson et al., 2013). Adverse childhood experiences can influence these developmental outcomes (O’Donnell et al., 2014). Like many aspects of behavior, development of attention and behavioral inhibition is driven by genetic makeup in interplay with environmental context and challenges in life. Exactly how behavioral inhibition and impulsivity are shaped by the environment, both early in life and throughout development, can be studied in preclinical models that allow precise control over the environment. However, the literature on this subject is surprisingly scarce. We therefore set out to examine the effect of a complex environment during adolescence –whether primed by early life adversity or not- on behavioral inhibition and impulsivity in male rats.

To measure impulsive action and inhibition of behavior in rats, we used the 5-choice serial reaction time task (5-CSRTT) (Robbins, 2002). This task is based on the continuous performance task in humans and was originally developed to measure sustained and divided attention. Animals are trained to respond to a visual stimulus appearing in one of five holes (divided attention) to obtain a reward. From trial to trial the stimulus light is randomly presented and the rat has to sustain attention over 100 trials. Rats have to learn to inhibit responding until a next visual stimulus is presented. Anticipatory responses prior to the presentation of the stimulus are punished with a time-out period (reward delay). These premature responses are regarded as a measure of impulsivity (Bari et al., 2008). After extensive training to acquire baseline levels of responding, either attentional load can be increased or behavioral inhibition can be challenged.

As the rodent approximation of our complex society, we housed animals post-weaning in an enriched laboratory housing, which is widely used as an experimental condition to provide a more complex environment to the animal compared to standard housing. The enriched laboratory housing is thought to more closely resemble the natural habitat or at least provides an environment where natural behaviors can be expressed (Wurbel, 2001). Environmental enrichment is a combination of complex inanimate and social stimulation, with the opportunity to exercise on a running wheel. Each of these components is necessary for effects of the enrichment to appear on most neural and behavioral parameters (Rosenzweig and Bennett, 1996; van Praag et al., 2000; Solinas et al., 2010), although behavioral adaptations might strongly depend on the exact nature of the stimulation.

It has been shown that environmental enrichment induces a range of structural changes in the brain, including an increased number of neurons, synapses, and dendritic branches especially in the cortex and hippocampal formation, resulting in improved learning and memory (van Praag et al., 2000; Halperin and Healey, 2011). These cognitive improvements are thought to arise from the arousal response when confronted with novelty and environmental complexity and mediated by cellular mechanisms underlying learning processes (van Praag et al., 2000). It has also been suggested that environmental enrichment provides a protective phenotype for drug abuse vulnerability (Stairs and Bardo, 2009; Zhang et al., 2014). This latter finding might provide a link to behavioral inhibition, since impulsivity (lack of inhibition) is thought to be a risk factor for abuse vulnerability (Perry and Carroll, 2008; Dalley et al., 2011).

Early life manipulations in rodent models and their long-lasting priming consequences for behavior and neuroendocrine function have been extensively studied (Meaney, 2010; Claessens et al., 2011; Haller et al., 2014). For instance, single, or repeated separation of newborn pups from their dam for a certain time period has been shown to induce stress hyper responsiveness, increased emotionality and impaired cognitive performance (Pryce and Feldon, 2003; Levine, 2005). Early postnatal maternal separation can also impact on prefrontal cortex functioning and the mesocortical dopamine system, both important in behavioral inhibition (Sullivan and Brake, 2003). Studies on early life influences on impulsivity are scarce, although there is some evidence of increased risk taking behavior in adolescence after maternal separation (Colorado et al., 2006; Spivey et al., 2009). In an extreme case of separation, rat pups that were individually housed and artificially reared were shown to be more impulsive (Lovic et al., 2011).

The aim of the present study was threefold. First, we wanted to test whether a complex rearing environment influences behavioral inhibition and sustained attention in adulthood, using the 5-choice serial reaction time task. Secondly, we were interested to know how a prolonged maternal deprivation (MD) early in life (24 h on postnatal day 3) would impact on these same measures. Finally, we tested if the two manipulations interact, showing counteractive or additive effects.

## Materials and Methods

### Animals

Male and female Wistar rats were obtained at 6 weeks of age (Charles River Laboratories, Arbresle, France). Animals were kept in a temperature (21°C) and humidity (55%) controlled room with a 12 h light–dark cycle (lights on at 7:00 am). Breeding started after animals had been familiarized with our animal facility for at least 3 weeks. Food and water was available ad libitum. For this experiment, we used the offspring of 14 dams, equally distributed over the experimental groups. Only male offspring (*n* = 48) was used for testing. Testing started at 90 days of age when animals weighed on average 330 grams. Three weeks before testing, the light-dark cycle was reversed (lights off at 7:00 am) to assure that animals were tested in their active phase. One week before testing males were gradually food deprived (16 grams of chow a day) until they reached 90–95% of their free-fed weight and they were kept within this range throughout testing. Once a week cages were changed, and general health status was checked. Experiments were approved by the local committee for Animal Health, Ethics and Research of Utrecht University. Animal care was conducted in accordance with the EC Council Directive of November 1986 (86/609/EEC).

### Early Life Experience: Breeding and Maternal Deprivation

Two females were paired with a male for 10 days. After removing the male, the two females stayed together for another week and were then individually housed to prepare for birth. A paper towel was provided to the mothers to supplement nesting material. At postnatal day 3, dams were taken out of their home cage and placed in a new cage. Only litters with a minimum of six pups were retained and large litters were culled to a maximum of 10 pups taking gender balance into consideration (sex ratio after culling: 5/5 or 4/6). Mothers in the control group were placed back into their home cage within 2 min, while for the experimental group MD started. During maternal separation, litters stayed together in their home cage and were transported to an adjacent room. The cage was placed on a heating plate to prevent hypothermia of the pups. After 24 h they were taken back to the original room and the mother was reunited with her litter.

### Adolescent Experience: Weaning and Complex Housing Environment

Pups were weaned at 21 days of age and were housed in (same-sex) pairs in standard makrolon cages (37 × 20 × 18 cm). Animals in the complex condition were group-housed following weaning (10–11 animals per cage), and after a week transferred to Marlau^TM^ cages (Viewpoint, Lyon, France). These cages (60 × 80 × 51 cm) have two floors and provide a complex and challenging environment for the rats (Fares et al., 2013). The first floor contains a big compartment with three running wheels, a shelter, ad libitum access to water, and a climbing ladder to the second floor, where a maze has to be passed to gain access to a tubing leading to the food compartment on the first floor. Via a one-way passage rats could regain access to the bigger first floor compartment. Before adult testing started, the maze was changed three times a week (alternating between 12 different configurations), assuring novelty and sustained cognitive stimulation. Territorial dominance was avoided by the presence of two gates on each side of the maze. To avoid disturbance during the daily 5-choice sessions, mazes were changed only once a week in adulthood, from the start of testing.

In many studies the enriched environment is not compared to standard housing, but to isolation rearing in which adolescent animals are completely deprived of social contact. This is a stressful condition in itself, and thus of limited value as a control condition for the effects of a complex environment (Hall, 1998; Van den Buuse et al., 2003; Solinas et al., 2010). Therefore, we used standard laboratory housing as control condition. Pups from the same mother were placed in both the experimental (complex housing) and control group (standard housing) to minimize litter effect. No more than two pups from 1 mother were placed in an experimental condition.

### Testing: 5-Choice Serial Reaction Time Task (5-CSRTT)

The following groups of animals were tested in the 5-choice serial reaction time task: Animals in the standard housing condition, either non-maternally deprived (standard housing no-MD, *n* = 8) or maternally deprived (standard housing MD, *n* = 8) and animals in the complex housing condition, either non-maternally deprived (complex housing no-MD *n* = 16) or maternally deprived (complex housing MD, *n* = 16). Daily sessions were performed during the dark phase (Monday–Friday), using procedures adapted from (Robbins, 2002) and (Bari et al., 2008).

#### Apparatus

The 5-CSRTT was conducted in operant conditioning chambers (Med Associates, St. Albans, VT, USA). Each chamber (30.5 cm × 24.1 cm × 21 cm) was located within a larger exterior opaque box equipped with exhaust fans that assured air renewal and masked background noise. The rear wall of the chamber was curved and contained a set of five holes, each equipped with an infrared detector and a yellow light emitting diode stimulus light. Sucrose pellets (45 mg, Formula P; Bio-Serv) were delivered at the opposite wall, in a larger pellet magazine, also equipped with infrared detectors. A white house light, located at the roof, could be switched on. Experimental contingencies were controlled and data were collected using MED-PC version 14.0 (Med Associates).

#### Habituation and Pellet Magazine Training

During the first 2 days, animals were habituated to the chambers for 20 min. Sucrose pellets were placed in all five response holes and in the pellet magazine. Habituation was followed by two magazine training sessions, where 80 sucrose pellets were delivered in the pellet magazine within 20 min., with an average interval of 15 s.

#### Training 5-Choice Task

Rats were trained to respond to a brief visual stimulus presented randomly in one of the five nose poke apertures to obtain a sucrose pellet. Each training session started with a 2 min. habituation period in which no reward could be obtained (house light switched on). Then the house light was switched off, a ‘free’ pellet was given, and the rat initiated the first trial by collecting this pellet in the pellet hole. On the start of a trial, one stimulus hole was illuminated. With a nose entry into this hole, a sucrose pellet was released into the pellet hole. After collecting this pellet an inter-trial-interval of 5 s (ITI5) started, followed by the next trial. A session ended when 100 trials had been accomplished or 30 min had elapsed. In *phase I of training*, all five stimulus lights were ‘ON’ at the start of a trial and a nose entry in either hole released a sucrose pellet. Animals were trained until each rat obtained 100 pellets (all within 4 days). Starting *phase II of training*, stimulus holes were illuminated in a pseudorandom order and each hole was illuminated 20 times during a 100 trials session. In phase II of training a stimulus hole was illuminated until nose entry. Entries in other (unlit) holes were counted, but without consequences. All animals obtained 100 pellets within 3 days. In *phase III of training* stimulus time was gradually decreased (16, 8, 4, 2, 1.5, 1.2 s) to reach the training endpoint of 1.2 s. The rats had a limited time to respond to the stimulus (limited hold = stimulus time + 2 s., with a minimum of 5 s). In this stage of training, an omission (no response), premature response (response in ITI) or incorrect response (response in unlit hole) resulted in a time-out period. During this time-out period, no reward could be obtained (house light switched on). Responding in stimulus holes during time-out resulted in a reset of the time-out period. Animals were trained on each stimulus duration until they finished 100 trials in 30 min with a performance accuracy >80% (correct choice) and errors of omission <20. Training was completed when animals reached this stable baseline responding at 1.2 s stimulus duration over at least three consecutive training days. Apart from habituation and time-out periods, the house light was switched off during the test in order to increase the contrast for visual discrimination of the stimulus lights for the albino Wistar rat.

#### Behavioral Control and Attention in the 5-Choice Task

Behavioral control, i.e., the ability to withhold responding, was challenged in two ways: (1) Lengthening the ITI to 7 s and (2) using a random ITI (5, 7, 10, 13, and 15 s). Attention was investigated by (1) increasing attentional load by shortening the stimulus time to 0.5 sec or (2) testing selective attention by introducing a novel object in the cage. This object was a wooden block of 3 cm high covering the middle line of the chamber and providing a light hurdle between stimulus holes and pellet hole. Between test sessions, baseline sessions (ITI5, 1.2 s stimulus duration) were performed until stable responding was resumed. The following measures were recorded (1) *Accuracy*: Percentage of correct responses [(correct/correct + incorrect)×100] (2) *Omissions:* Number of missed trials (3) *Latency to correct*: Latency between stimulus presentation and correct choice (4) L*atency to reward:* Latency to collect the reward after correct choice (5) *Premature responses*: Number of nose pokes before the presentation of the stimulus light (6) *Perseverative responses*: Number of nose pokes after correct choice. Additional behavior in no-reward periods was recorded: (7) Number of nose pokes in the pellet hole during inter-trial intervals (NP pellet hole ITI) and frequency of nose pokes in pellet hole and stimulus holes, respectively, per time-out period (NP pellet hole/TO and NP stimulus holes/TO). Since the number of time-out periods varied between animals, behavior during this “punishment” period was computed as behavior per time-out.

#### Statistical Analyses

All statistical analyses were performed using SPSS for windows version 21. Outlying scores (>3.29 SD above the mean), were substituted with the next highest score [winsorized, (Tabachnick and Fidell, 2006)] to mitigate excessive influence of outliers without excluding subjects. In total five data points were winsorized (in five different measures). Of note, if these five outliers were excluded, this did not change any of the outcomes.

In all analyses, housing condition (standard vs. complex) and early life experience (no-MD vs. MD) served as between-subject factors. To compare experimental groups on habituation, repeated measures ANOVAs were performed over 5 min blocks. Univariate ANOVAs were performed to compare group differences at the end of training. Responses in the testing conditions (ITI 7 s, ITI random, stim 0.5 s and novel object) were compared to responses during baseline (end of training) in repeated measures ANOVAs. We report both *p*-values and effect size ($\eta_{p}^{2}$).

Not all animals reached 100 trials in the ITI7 and random ITI tests. Responses were therefore also calculated per trial. Performance per trial was highly correlated with total performance (*r*s ranging between 0.83 and 0.99, *p* < 0.001), and results of the analyses were similar. We therefore report on total performance.

## Results

### Bodyweight

**FIGURE 1:**
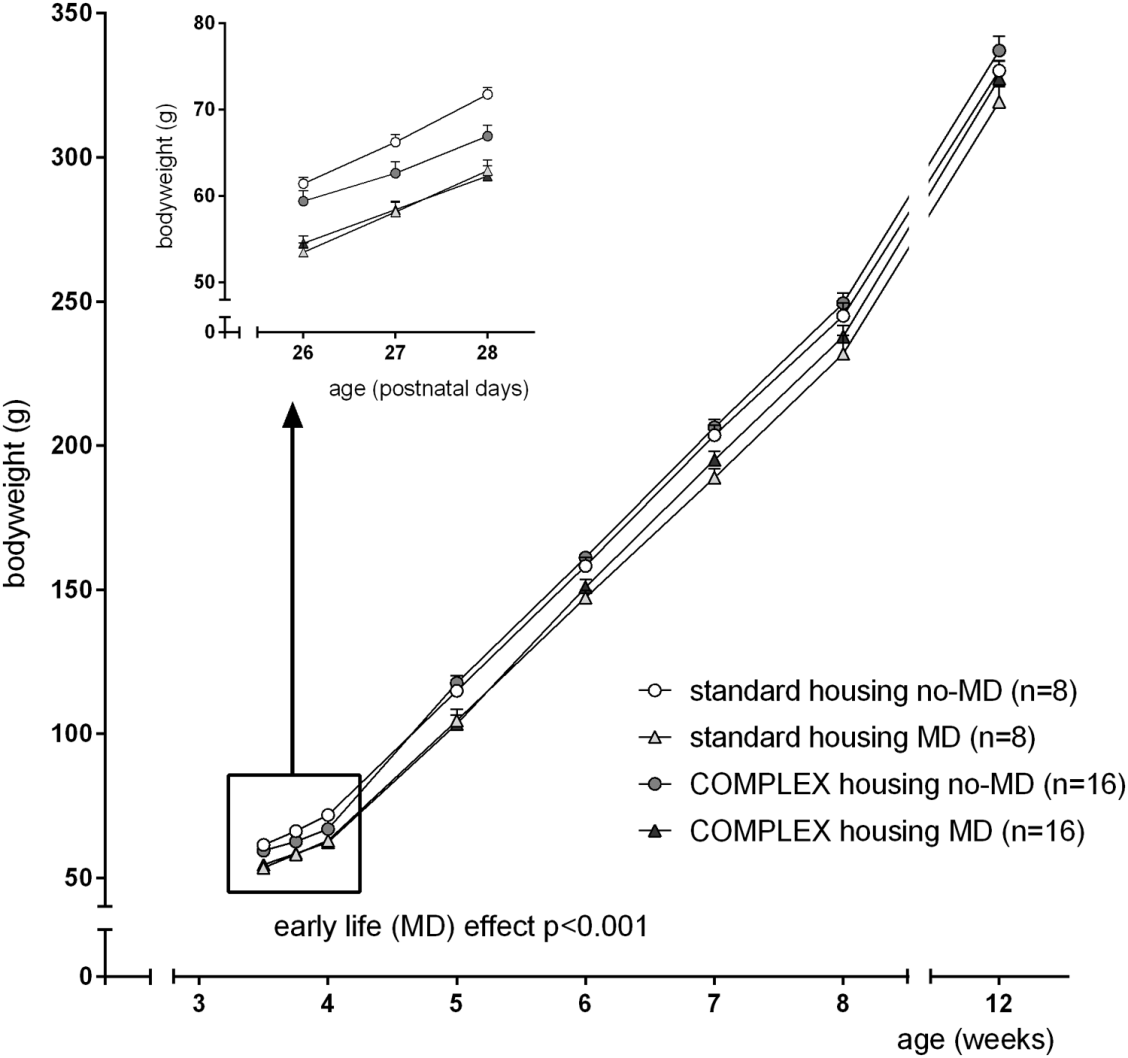
**The effect of complex housing and early life maternal deprivation (MD) on bodyweight.** First measure at postnatal day 26 (PND 26), when animals either entered the complex home cage or stayed in the standard home cage. Last measure at 12 weeks, just before the start of the 5-choice experiment. Data represent mean ± SEM. Standard housing no-MD (*n* = 8) and MD (*n* = 8), complex housing no-MD (*n* = 16), and MD (*n* = 16).

After weaning, and before the start of complex housing (at PND26), maternally deprived animals had a lower bodyweight compared to non-deprived animals [*F*(1,47) = 30.1, *p* < 0.001, $\eta_{p}^{2}$ = 0.41]. This difference in bodyweight continued over several weeks [4–8 weeks: *F*(1,44) = 16.2, *p* < 0.001, $\eta_{p}^{2}$ = 0.27], but was not significant anymore at the start of 5-choice testing [*F*(1,47) = 2.83, *p* = 0.10]. The 1st week following the change in housing condition, complex housing caused a transient decline in bodyweight [time^∗^housing effect: *F*(3,42) = 8.6, *p* < 0.001, $\eta_{p}^{2}$ = 0.38], that was not visible anymore at 5 weeks of age (**Figure 1**).

### Habituation to the 5-Choice Chamber and Speed of Learning During Training

**FIGURE 2:**
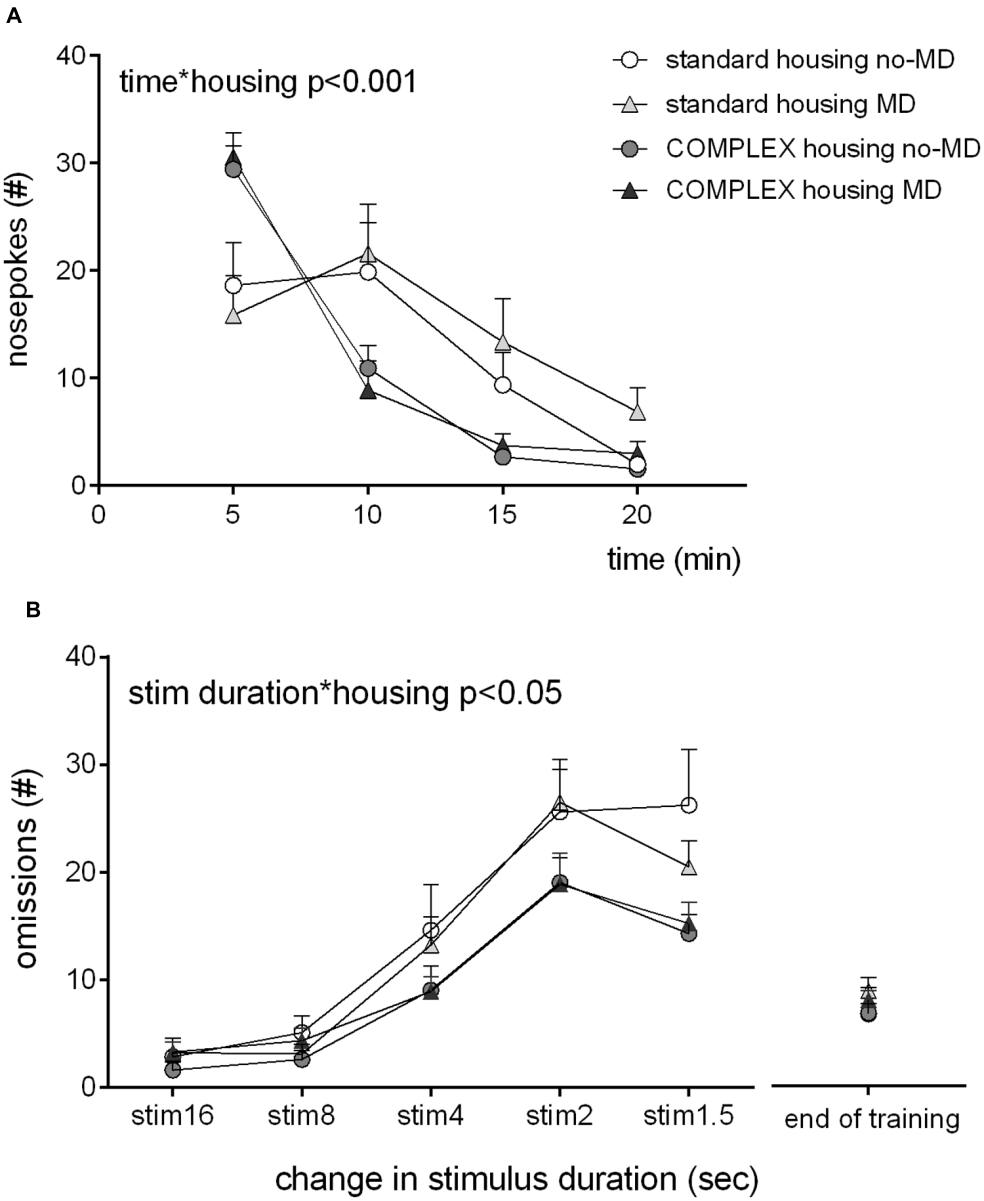
**The effect of complex housing and early life MD on (A) habituation to the 5-choice SRTT chambers as expressed by the number of nose hole entries in blocks of 5 min and (B) speed of learning the 5-choice SRTT as expressed by the number of omissions at the start of each change (decrease) in stimulus duration.** Note that at end of training (at stim duration 1.2 s) no group differences exist. Graphs represent mean ± SEM. Standard housing no-MD (*n* = 8) and MD (*n* = 8), complex housing no-MD (*n* = 16) and MD (*n* = 16).

All animals habituated to the test chamber (**Figure 2A**), as evident from a main effect of time, *F*(3,42) = 69.72, *p* < 0.001, $\eta_{p}^{2}$ = 0.83, over the 20 min of test duration. All groups showed a decrease in number of nose pokes in the five nose poke holes. Housing condition was related to habituation [time^∗^housing *F*(3,42) = 12.4, *p* < 0.001, $\eta_{p}^{2}$ = 0.47], the complex housed animals habituated faster, particularly in the first interval (0–10 min) [time^∗^housing *F*(1,44) = 21.29, *p* < 0.001, $\eta_{p}^{2}$ = 0.33]. MD did not influence habituation [time^∗^early life *F*(3,42) = 0.84, *p* = 0.48, $\eta_{p}^{2}$ = 0.06], and did not moderate the effect of housing condition [time^∗^early life^∗^housing *F*(3,42) = 0.43, *p* = 0.73, $\eta_{p}^{2}$ = 0.03].

Speed of learning was assessed by comparison of the 1st days on which the stimulus light decreased in duration during training phase III [16 to 1.5 s, **Figure 2B**). The number of omissions increased when stimulus duration was shortened [*F*(4,41) = 58, *p* < 0.001, $\eta_{p}^{2}$ = 0.85]. Housing condition influenced this increase [stim duration^∗^housing *F*(4,41) = 3.15, *p* = 0.024, $\eta_{p}^{2}$ = 0.24]: the complex housed animals made fewer omissions compared to the standard housed animals, hence learned the task faster. MD did not influence speed of learning [stim duration^∗^early life *F*(4,41) = 0.79, *p* = 0.54, $\eta_{p}^{2}$ = 0.07], or moderate the effect of housing [stim duration^∗^early life^∗^housing *F*(4,41) = 1.00, *p* = 0.42, $\eta_{p}^{2}$ = 0.09]. No differences in accuracy were observed (data not shown).

### Performance at End of Training (Baseline)

At the end of training, all animals finished 100 trials within 30 min (duration *M* = 18.5–18.9 min) and reached the criteria for learning with an accuracy >80% (*M* = 92.6–94.7%) and omissions <20 (*M* = 7.6–10.0) (see **Table 1**). Numbers of premature (*M* = 4.8–8.4) and perseverant responses (*M* = 4.0–6.3) were low. Neither housing condition, nor MD influenced performance on these measures at the end of the training period. There was, however, a main effect of housing condition on the latency to respond to the correct stimulus [*F*(1,47) = 6.63, *p* = 0.013, $\eta_{p}^{2}$ = 0.13]. Thus, complex housed animals responded faster. No differences were observed in the latency to collect the reward.

**Table 1:** The effect of complex housing and early life maternal deprivation (MD) on performance in the 5-choice SRTT at end of training (baseline).

	No-MD		MD	
	Standard	Complex	Standard	Complex
Accuracy(%)	94,71 ± 1,42	92,67 ± 1,04	94,63 ± 1,06	92,87 ± 1,21
Omissions(#)	7,58 ± 2,75	7,69 ± 1,01	10,04 ± 1,40	9,06 ± 0,96
Premature(#)	5,75 ± 1,56	8,35 ± 1,25	4,83 ± 1,06	6,75 ± 0,88
Perseverant(#)	4,54 ± 1,35	4,13 ± 0,77	6,29 ± 1,24	4,04 ± 0,54
Latency to correct(sec) ^a∗^	0,73 ± 0,04	0,61 ± 0,01	0,68 ± 0,03	0,66 ± 0,03
Latency to reward (sec)	1,30 ± 0,09	1,33 ± 0,06	1,23 ± 0,08	1,25 ± 0,04
NP pellet hole ITI (#)^a∗^	280,50 ± 59,14	132,75 ± 23,21	228,75 ± 25,69	170,69 ± 44,81
NP pellet hole/TO (#)^a∗^	2,35 ± 0,26	1,47 ± 0,20	2,22 ± 0,28	1,93 ± 0,27
NP stimulus holes/TO (#)^a∗∗^	0,23 ± 0,07	0,39 ± 0,03	0,24 ± 0,04	0,38 ± 0,04
total duration (min)	18,58 ± 0,48	18,92 ± 0,25	18,54 ± 0,34	18,83 ± 0,25

*General performance and stimulus (stimulus holes) and goal (pellet hole) approach in no-reward periods (NP, nose pokes; ITI, inter trial interval, and TO, time-out). Standard housing no-MD (n = 8) and MD (n = 8), complex housing no-MD (n = 16), and MD (n = 16). ^a^main effect of housing ^∗^p < 0.05, ^∗∗^p < 0.01.*

An effect of complex housing was also seen in the time-out periods, when no reward could be obtained. Animals in the complex housing condition approached the stimulus holes more often compared to animals in the standard housing [*F*(1,47) = 10.4, *p* < 0.01, $\eta_{p}^{2}$ = 0.19] and the pellet hole less often [*F*(1,47) = 4.72, *p* = 0.035, $\eta_{p}^{2}$ = 0.10]. Moreover, pellet hole visits in the inter-trial interval periods were also affected by housing [*F*(1,47) = 5.89, *p* = 0.019, $\eta_{p}^{2}$ = 0.12], showing again fewer visits to the pellet hole in the complex housed versus the standard housed animals. Thus, the complex housed animals showed features of sign trackers (more visits to the stimulus holes) and were faster in responding to the stimulus. However, all animals equally performed at the end of training in terms of omission, accuracy and total test duration (acquisition criteria).

### Behavioral Control I: Prolonged Inter-Trial Interval (ITI 7 s)

**FIGURE 3:**
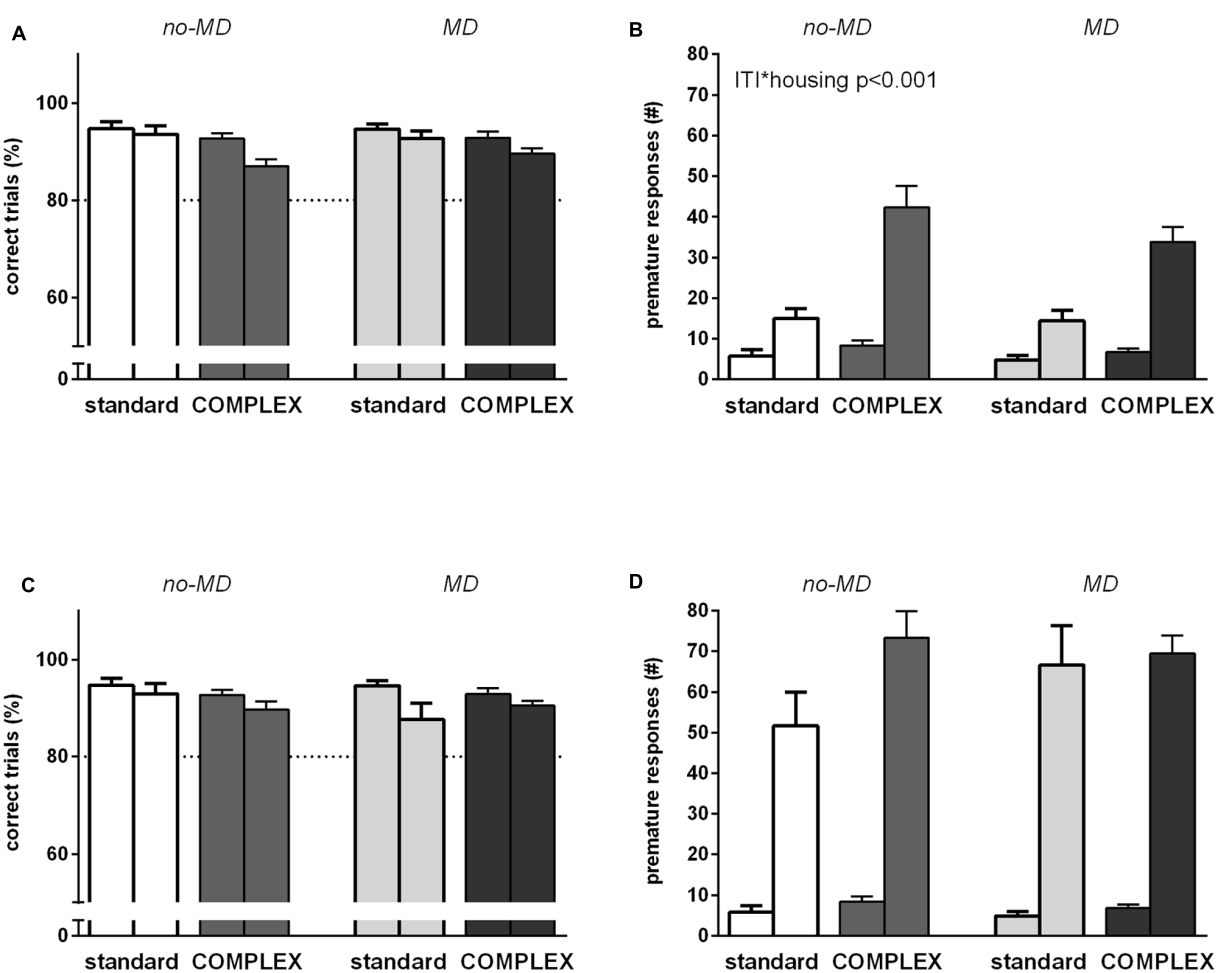
**The effect of complex housing and early life MD on performance in the 5-choice SRTT when *behavioral inhibition* is challenged.** First bar of each color represents baseline performance, second bar of each color represents test performance. Accuracy (% correct trials) and number of premature responses when **(A,B)** the inter trial interval (ITI) is prolonged to 7 s or **(C,D)** under a randomized inter trial interval protocol. Graphs represent mean ± SEM. Dotted horizontal lines represent acquisition criteria. Standard housing no-MD (*n* = 8) and MD (*n* = 8), complex housing no-MD (*n* = 16) and MD (*n* = 16).

Overall, accuracy decreased [*F*(1,44) = 13.2, *p* < 0.01, $\eta_{p}^{2}$ = 0.23] and premature responses increased [*F*(1,44) = 88.1, *p* < 0.001, $\eta_{p}^{2}$ = 0.67] in response to an increase in inter-trial interval (**Figures 3A,B**). Animals living in a complex environment responded differently to the 7 s inter-trial interval compared to the animals in the standard housing condition in terms of premature responses [ITI^∗^housing *F*(1,44) = 24.7, *p* < 0.001, $\eta_{p}^{2}$ = 0.36], but not accuracy [ITI^∗^housing *F*(1,44) = 3.09, *p* = 0.086, $\eta_{p}^{2}$ = 0.07]. A stronger increase in premature responses was seen in the complex housed animals. MD did not influence responding to an ITI 7 s challenge in either accuracy [ITI^∗^early life *F*(1,44) = 0.23, *p* = 0.64, $\eta_{p}^{2}$ = 0.005] or premature responses [ITI^∗^early life *F*(1,44) = 0.60, *p* = 0.44, $\eta_{p}^{2}$ = 0.01]. Moreover, MD did not moderate the effect of housing on accuracy [ITI^∗^early life^∗^housing *F*(1,44) = 0.94, *p* = 0.34, $\eta_{p}^{2}$ = 0.02] nor on premature responses [ITI^∗^early life^∗^housing *F*(1,44) = 0.71, *p* = 0.40, $\eta_{p}^{2}$ = 0.02]. Animals in the complex housing condition thus showed poorer behavioral inhibition compared to animals in the standard housing condition.

### Behavioral Control II: Random Inter-Trial Interval (ITI 5, 7, 10, 13, and 15 s)

Accuracy decreased [*F*(1,44) = 10.6, *p* < 0.01, $\eta_{p}^{2}$ = 0.20] and premature responses increased dramatically [*F*(1,44) = 312.8, *p* < 0.001, $\eta_{p}^{2}$ = 0.88] in response to random inter-trial intervals (**Figures 3C,D**). Overall, the latency to correct response increased [*F*(1,44) = 44.9, *p* < 0.001, $\eta_{p}^{2}$ = 0.51] and the latency to reward pick-up decreased [latency to reward *F*(1,44) = 39.2, *p* < 0.001, $\eta_{p}^{2}$ = 0.47], meaning the animals were slower in responding to the correct stimulus, but quicker to collect the sugar pellet, once they poked in the correct stimulus hole (data not shown). This test was very challenging for all animals and neither housing condition nor MD significantly influenced responding in terms of accuracy [ITI^∗^housing *F*(1,44) = 0.59, *p* = 0.45, $\eta_{p}^{2}$ = 0.01; ITI^∗^early life *F*(1,44) = 1.14, *p* = 0.29, $\eta_{p}^{2}$ = 0.03; ITI^∗^early life^∗^housing *F*(1,44) = 1.81, *p* = 0.19, $\eta_{p}^{2}$ = 0.04] or premature responses [ITI^∗^housing *F*(1,44) = 2.26, *p* = 0.14, $\eta_{p}^{2}$ = 0.05; ITI^∗^early life *F*(1,44) = 1.05, *p* = 0.31, $\eta_{p}^{2}$ = 0.02; ITI^∗^early life^∗^housing *F*(1,44) = 1.87, *p* = 0.18, $\eta_{p}^{2}$ = 0.04].

### Attention I: Short Stimulus Duration (0.5 s)

**FIGURE 4:**
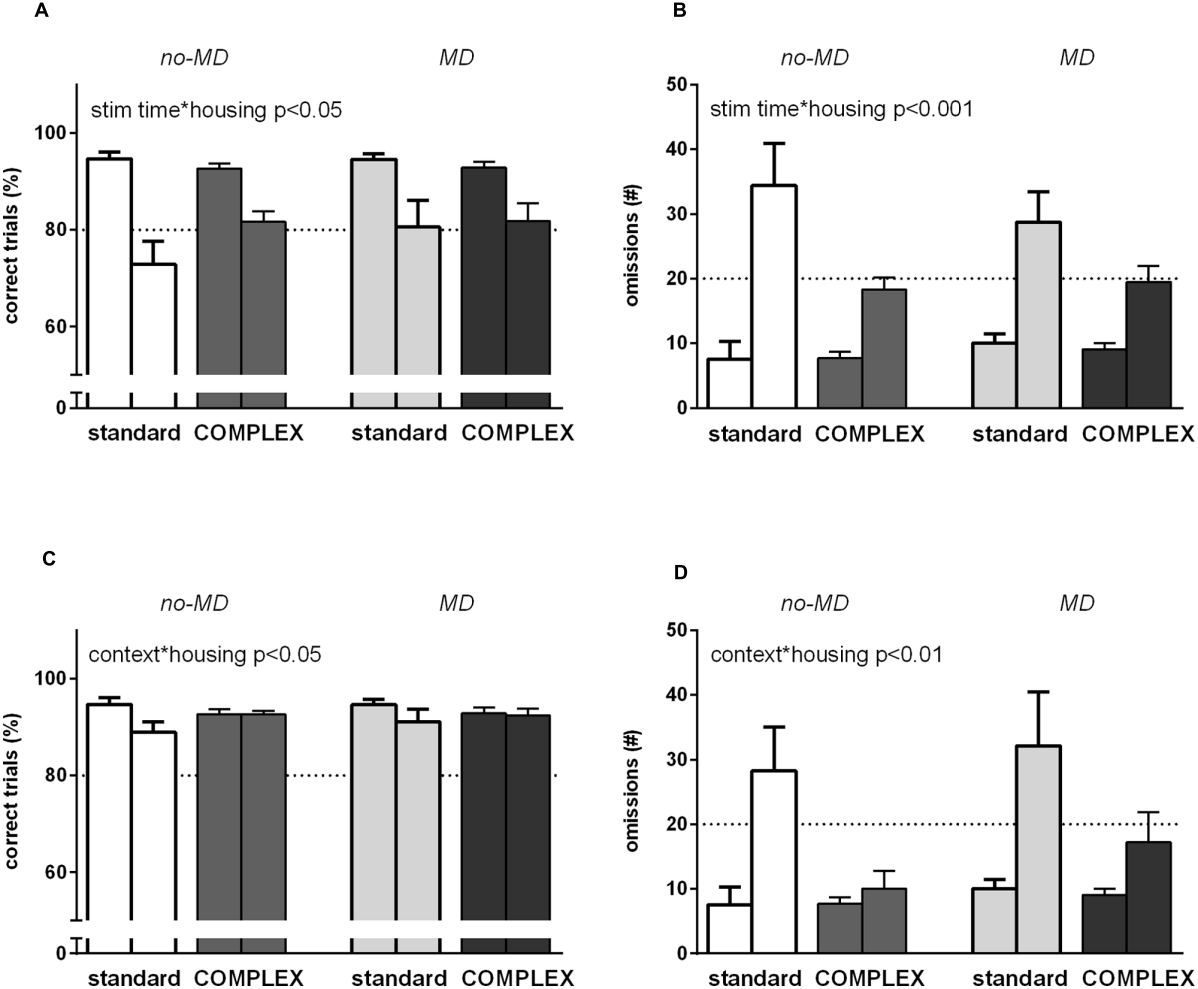
**The effect of complex housing and early life MD on performance in the 5-choice SRTT when *attentional load* is increased.** First bar of each color represents baseline performance, second bar of each color represents test performance. Accuracy (% correct trials) and number of omissions when **(A,B)** the stimulus duration is shortened to 0.5 s or **(C,D)** when a novel object is introduced in the cage. Graphs represent mean ± SEM. Dotted horizontal lines represent acquisition criteria. Standard housing no-MD (*n* = 8) and MD (*n* = 8), complex housing no-MD (*n* = 16), and MD (*n* = 16).

A strong decrease in accuracy [*F*(1,44) = 77.1, *p* < 0.001, $\eta_{p}^{2}$ = 0.64] and a strong increase in omissions [*F*(1,44) = 107.3, *p* < 0.001, $\eta_{p}^{2}$ = 0.71]were observed in response to a shorter stimulus duration (**Figures 4A,B**). Animals became quicker when stimulus duration decreased as apparent from a decrease in both latency to correct [*F*(1,44) = 47.2, *p* < 0.001, $\eta_{p}^{2}$ = 0.52] and, to a lesser extent, latency to reward [*F*(1,44) = 8.68, *p* < 0.01, $\eta_{p}^{2}$ = 0.17] (data not shown).

Animals living in a complex housing environment differed in responding to a short stimulus from the standard housed animals in accuracy [stim time^∗^housing *F*(1,44) = 4.39, *p* = 0.042, $\eta_{p}^{2}$ = 0.091] and number of omissions [stim time^∗^housing *F*(1,44) = 14.5, *p* < 0.001, $\eta_{p}^{2}$ = 0.25]. A stronger decrease in accuracy and a stronger increase in omissions was seen for the animals in the standard vs. the complex housing environment. MD did not influence responding to a short stimulus challenge in either accuracy [stim time^∗^early life *F*(1,44) = 1.39, *p* = 0.25, $\eta_{p}^{2}$ = 0.03] or number of omissions [stim time^∗^early life *F*(1,44) = 1.66, *p* = 0.21, $\eta_{p}^{2}$ = 0.04]. Moreover, it did not moderate the effect of housing condition on accuracy [stim time^∗^early life^∗^housing *F*(1,44) = 1.40, *p* = 0.24, $\eta_{p}^{2}$ = 0.03] or number of omissions [stim time^∗^early life^∗^housing *F*(1,44) = 1.51, *p* = 0.23, $\eta_{p}^{2}$ = 0.03]. Thus, compared to the standard housed animals, animals in the complex housing condition performed better in this task and showed more sustained attention.

### Attention II: Introducing a Novel Object (Woodblock)

When a woodblock was introduced in the 5-choice chamber, accuracy decreased [*F*(1,44) = 7.66, *p* < 0.01, $\eta_{p}^{2}$ = 0.15] and more omissions were made [*F*(1,44) = 23.6, *p* < 0.001, $\eta_{p}^{2}$ = 0.35] (**Figures 4C,D**). Latency to correct response and latency to reward both increased (data not shown), with the strongest increase in latency to reward [latency to correct *F*(1,44) = 11.1, *p* < 0.01, $\eta_{p}^{2}$ = 0.20; latency to reward *F*(1,44) = 71.9, *p* < 0.001, $\eta_{p}^{2}$ = 0.62].

Animals living in a complex housing environment differed from the standard housing condition in accuracy [context^∗^housing *F*(1,44) = 6.16, *p* = 0.017, $\eta_{p}^{2}$ = 0.12) and in number of omissions [context^∗^housing *F*(1, 44) = 8.67, *p* < 0.01, $\eta_{p}^{2}$ = 0.17]. The standard housed animals decreased in accuracy while the complex housed animals did not, and standard housed animals showed a stronger increase in omissions. MD did not influence responding when context changed by introducing a woodblock in either accuracy [context^∗^early life *F*(1,44) = 0.30, *p* = 0.59, $\eta_{p}^{2}$ = 0.007] or number of omissions [context^∗^early life *F*(1,44) = 0.43, *p* = 0.52, $\eta_{p}^{2}$ = 0.01]. Moreover, MD did not moderate the effect of housing condition on accuracy [context^∗^early life^∗^housing *F*(1,44) = 0.55, *p* = 0.46, $\eta_{p}^{2}$ = 0.012] or number of omissions [context^∗^early life^∗^housing *F*(1,44) = 0.16, *p* = 0.69, $\eta_{p}^{2}$ = 0.004]. Also in this attention task, animals in the complex housing condition performed better compared to animals in the standard housing condition.

## Discussion

We showed that a complex rearing environment improves speed of learning in the 5-choice task. Eventually, though, animals reared in standard housing also acquired the learning criteria and baseline performance was similar for all experimental groups. Animals in the complex housing condition subsequently performed better when stimulus duration was shortened or a novel object was introduced in the cage, showing higher levels of attention. However, behavioral inhibition was reduced in these animals when challenged with a longer inter-trial interval. MD did not influence responding in the 5-choice task, nor did it moderate the effects of adolescent complex housing.

### Effects of Complex Housing Condition

The start of complex housing caused an initial drop in bodyweight, though animals recovered quickly. When exposed for the first time to the 5-choice test chambers, complex housed animals started to explore the environment more intensively than standard housed animals, but they also habituated rapidly to the new chambers. This is in line with results of Zimmermann et al. (2001), showing faster habituation to novelty in rats provided with a large near-to-natural complex rearing environment. A faster acquisition of spatial information in complex housed animals (Leggio et al., 2005) might explain the faster habituation to a new (spatial) context. This might also have influenced performance when a wood block was introduced in the cage, allowing attention to be quickly allocated again to the stimulus lights instead of being distracted by the novel object. More efficient processing of spatial information and better strategies to deal with environmental challenges most likely are the result of the complex rearing environment that provides novelty and sustained cognitive stimulation in a complex social setting. Faster learning of the 5-choice SRTT and better performance when attention is challenged thus probably result from changes in alerting, orienting and executive function networks, which are all involved in attention (Petersen and Posner, 2012).

Complex housing might more efficiently wire functional brain networks of attention, but this seems to come at a price. When behavioral inhibition is challenged, complex housed animals show more premature responses and thus –within the context of the 5-choice task– perform worse. There could be a trade-off between a quick response on the one hand and inhibition of behavior on the other hand in an environment that makes it profitable to be fast. The alerting network, though partly overlapping in brain circuitry with attentional networks, is more strongly driven by noradrenergic input from the locus coeruleus to the frontal and parietal lobes, whereas impulsivity is more closely linked to a dopaminergic system involving the basal ganglia, anterior cingulate and the top–down control exerted by cortical, especially prefrontal circuitry (Dalley et al., 2011; Petersen and Posner, 2012). Complex housing might influence both networks. Neuronal changes have indeed been observed in the medial prefrontal cortex and mesocorticolimbic structures of complex housed animals, in addition to changes in neurotransmission, including altered dopaminergic functioning (Stairs and Bardo, 2009).

We show that complex housed animals are more impulsive in the 5-choice SRTT. This is in apparent contrast with a study that showed less impulsivity in enriched housed animals in an adjusting delay task (Perry et al., 2008). Impulsivity, however, is not a simple unitary construct (Winstanley et al., 2006; Dalley et al., 2008) and includes lack of inhibition of behavior, lack of reflection on the consequences of one’s acts, and delay aversion, with difficulties to postpone reward. In behavioral paradigms, impulsive choice and impulsive action can be studied separately. Impulsive choice (impulsive decision making) concerns actions that are initiated without much concern for other possible options or outcomes, while impulsive action involves lack of behavioral inhibition, including actions that are premature, mis-timed or difficult to suppress or control (Dalley et al., 2008). The 5-choice SRTT measures impulsive action, while the adjusting delay task measures impulsive choice. These measures might have independent underlying mechanisms (Winstanley et al., 2006), which can lead to apparently contrasting results. Furthermore, control animals in the impulsive choice study were socially isolated rather than standard housed, which might have influenced the results, since social isolation is stressful in itself.

After an experience of social isolation or chronic mild stress during adolescence, impulsivity in the 5-choice SRTT was earlier found to be increased compared to standard housed animals, while attention was not affected (Baarendse et al., 2013; Comeau et al., 2014). Together with other findings on social isolation rearing (Hall, 1998; Van den Buuse et al., 2003; Solinas et al., 2010), it appears that social isolation and chronic mild stress more generally disrupt cognitive control in adulthood, while complex housing animals might involve a trade-off between attention and impulsivity.

It has been suggested that environmental enrichment provides a protective phenotype for drug abuse vulnerability and leads to less impulsivity (Stairs and Bardo, 2009; Zhang et al., 2014). Indeed, rats that were highly impulsive in the 5-choice SRTT showed enhanced self-administration of cocaine, nicotine and sucrose compared to low impulsive rats and had reduced DA D2/3 receptor binding in the ventral striatum (Dalley et al., 2007). The increase in premature responses in these highly impulsive rats in an ITI7 challenge shows a striking similarity with the behavior of our complex housed animals, even though baseline premature responses are clearly lower in our animals. Like complex housed animals, impulsive rats also tended to be quicker to respond to the stimulus. This similarity would not be in favor of a drug protective phenotype in rats reared in a complex (“enriched”) environment.

We also found that during the no-reward periods (inter-trial interval and time-out), the complex housed animals preferably approached the stimulus holes rather than the sugar pellet compartment compared with standard housed animals. This fits with the description of sign trackers (Tunstall and Kearns, 2015). Thus, sign trackers primarily approach and contact the levers, while goal trackers primarily approach the site of food delivery. From an incentive salience point of view, the sign trackers attribute incentive salience to the conditioned stimulus, and they have indeed been shown to choose cocaine over food more often than goal trackers (Robinson et al., 2014; Tunstall and Kearns, 2015). We found reduced behavioral inhibition in the complex housed animals, who also presented characteristics of sign tracking animals. This would favor a drug prone over a drug protective phenotype. These findings raise the question whether the complex housed animals share the dopaminergic characteristics with highly impulsive rats. The high-attention high-impulsive phenotype could shed more light on the implication of impulsivity in drug abuse liability. Our data therefore raise the question to what extent ‘environmental enrichment’ is an appropriate term for continuous housing in a challenging environment in the presence of a relatively large group of conspecifics. We consider the designation ‘complex or challenging housing environment’ more appropriate.

### Effects of 24 h Maternal Deprivation

Following weaning, maternally deprived animals weighed less than control non-deprived animals, consistent with other studies (Burke et al., 2013; Marco et al., 2015). This difference continued for several weeks into young adulthood, but eventually disappeared. Based on studies applying 24 h MD on postnatal day 9 it has been proposed that disruption of leptin levels peaking around postnatal day 10 could explain bodyweight differences and altered metabolic programming (Marco et al., 2015). The sensitive window for these effects may not be limited to these postnatal days, since MD earlier in life seems to have similar disruptive effects.

Somewhat to our surprise, a quite severe early life stress of 24 h MD did not significantly influence learning or performance in the 5-choice task. Neither did we observe interactions of complex housing with the early life experience. The protocol of MD on postnatal day 3 has often been used in male rats and was shown to alter HPA axis activity (Rots et al., 1996; van Oers et al., 1997). Moreover, reduced levels of adult hippocampal neurogenesis were observed, paralleled by impaired spatial learning in the water maze, and improved contextual learning in fear conditioning (Oomen et al., 2010). Adaptation to early life stress might not always be unfavorable, as has also been shown in studies where animals with a history of early life stress perform poorly under low stress conditions, but outperform animals in stressful tasks (Champagne et al., 2008; Bagot et al., 2009; Oomen et al., 2010; Nederhof and Schmidt, 2012). Effects on information processing are not consistent. Twenty-four hour MDs performed at different ages did not influence adult sensorimotor gating in prepulse inhibition (Lehmann et al., 2000), although others found a reduced PPI in deprived animals (Ellenbroek et al., 1998). We extend the picture by showing no effect of MD at postnatal day 3 on attention and behavioral inhibition in the 5-choice task performed in adulthood. It remains to be tested whether deprivation at other time points might have differential effects.

Taken together, the present study suggests that living in a complex challenging environment, enriched in social stimuli, inanimate complexity, and cognitive stimulation, impacts on brain circuits implicated in both attention and behavioral inhibition. We observed a specific phenotype of improved attention, but impaired behavioral inhibition, as measured in the 5-choice SRTT. This points to a faster adaptation to changes in the environment, but at the expense of lower behavioral inhibition. The quite severe early life stress of 24 h MD did not by itself affect attention or behavioral control in the 5-choice SRTT, nor did it moderate the effects of complex housing. Timing and nature of early life adversity might be relevant for its influence on responses to later life environmental challenges in specific behavioral domains.

## Author Contributions

Authors have made substantial contributions to the following: Conception and design of the study: RV, MIJ, MB-K, MJ. Interpretation of data: RV, JK, MIJ, MB-K, MJ. Acquisition of data: RV, JK, LT, ML. Analysis of data: RV, JK, MIJ, MB-K, MJ. Drafting the article critically for important intellectual content: RV, MIJ, MB-K, MJ. Final approval of the version to be submitted: RV, JK, LT, ML, MIJ, MB-K, MJ. Agreement to be accountable for all aspects of the work in ensuring that questions related to the accuracy or integrity of any part of the work are appropriately investigated and resolved: RV, JK, LT, ML, MIJ, MB-K, MJ.

## Conflict of Interest Statement

The authors declare that the research was conducted in the absence of any commercial or financial relationships that could be construed as a potential conflict of interest.

## References

[B1] Baarendse P. J. Counotte D. S. O’Donnell P. Vanderschuren L. J. (2013). Early social experience is critical for the development of cognitive control and dopamine modulation of prefrontal cortex function. *Neuropsychopharmacology* 38 1485–1494. 10.1038/npp.2013.47 23403694 PMC3682143

[B2] Bagot R. C. van Hasselt F. N. Champagne D. L. Meaney M. J. Krugers H. J. Joels M. (2009). Maternal care determines rapid effects of stress mediators on synaptic plasticity in adult rat hippocampal dentate gyrus. *Neurobiol. Learn. Mem.* 92 292–300. 10.1016/j.nlm.2009.03.004 19292996

[B3] Bari A. Dalley J. W. Robbins T. W. (2008). The application of the 5-choice serial reaction time task for the assessment of visual attentional processes and impulse control in rats. *Nat Protoc.* 3 759–767. 10.1038/nprot.2008.41 18451784

[B4] Burke N. N. Llorente R. Marco E. M. Tong K. Finn D. P. Viveros M. P. (2013). Maternal deprivation is associated with sex-depend ent alterations in nociceptive behavior and neuroinflammatory mediators in the rat following peripheral nerve injury. *J. Pain* 14 1173–1184. 10.1016/j.jpain.2013.05.003 23850096

[B5] Champagne D. L. Bagot R. C. van Hasselt F. Ramakers G. Meaney M. J. de Kloet E. R. (2008). Maternal care and hippocampal plasticity: evidence for experience-dependent structural plasticity, altered synaptic functioning, and differential responsiveness to glucocorticoids and stress. *J. Neurosci.* 28 6037–6045. 10.1523/jneurosci.0526-08.2008 18524909 PMC6670331

[B6] Claessens S. E. Daskalakis N. P. van der Veen R. Oitzl M. S. de Kloet E. R. Champagne D. L. (2011). Development of individual differences in stress responsiveness: an overview of factors mediating the outcome of early life experiences. *Psychopharmacology (Berl.)* 214 141–154. 10.1007/s00213-010-2118-y 21165737 PMC3045508

[B7] Colorado R. A. Shumake J. Conejo N. M. Gonzalez-Pardo H. Gonzalez-Lima F. (2006). Effects of maternal separation, early handling, and standard facility rearing on orienting and impulsive behavior of adolescent rats. *Behav. Process.* 71 51–58. 10.1016/j.beproc.2005.09.007 16242858

[B8] Comeau W. L. Winstanley C. A. Weinberg J. (2014). Prenatal alcohol exposure and adolescent stress - unmasking persistent attentional deficits in rats. *Eur. J. Neurosci.* 40 3078–3095. 10.1111/ejn.12671 25059261 PMC4189965

[B9] Dalley J. W. Everitt B. J. Robbins T. W. (2011). Impulsivity, compulsivity, and top-down cognitive control. *Neuron* 69 680–694. 10.1016/j.neuron.2011.01.020 21338879

[B10] Dalley J. W. Fryer T. D. Brichard L. Robinson E. S. Theobald D. E. Laane K. (2007). Nucleus accumbens D2/3 receptors predict trait impulsivity and cocaine reinforcement. *Science* 315 1267–1270. 10.1126/science.1137073 17332411 PMC1892797

[B11] Dalley J. W. Mar A. C. Economidou D. Robbins T. W. (2008). Neurobehavioral mechanisms of impulsivity: fronto-striatal systems and functional neurochemistry. *Pharmacol. Biochem. Behav.* 90 250–260. 10.1016/j.pbb.2007.12.021 18272211

[B12] Ellenbroek B. A. van den Kroonenberg P. T. Cools A. R. (1998). The effects of an early stressful life event on sensorimotor gating in adult rats. *Schizophr. Res.* 30 251–260. 10.1016/S0920-9964(97)00149-7 9589519

[B13] Fares R. P. Belmeguenai A. Sanchez P. E. Kouchi H. Y. Bodennec J. Morales A. (2013). Standardized environmental enrichment supports enhanced brain plasticity in healthy rats and prevents cognitive impairment in epileptic rats. *PLoS ONE* 8:e53888 10.1371/journal.pone.0053888 PMC3544705 23342033

[B14] Fergusson D. M. Boden J. M. Horwood L. J. (2013). Childhood self-control and adult outcomes: results from a 30-year longitudinal study. *J. Am. Acad. Child Adolesc. Psychiatry* 52 709–717e701. 10.1016/j.jaac.2013.04.008 23800484

[B15] Hall F. S. (1998). Social deprivation of neonatal, adolescent, and adult rats has distinct neurochemical and behavioral consequences. *Crit. Rev. Neurobiol.* 12 129–162. 10.1615/CritRevNeurobiol.v12.i1-2.50 9444483

[B16] Haller J. Harold G. Sandi C. Neumann I. D. (2014). Effects of adverse early-life events on aggression and anti-social behaviours in animals and humans. *J. Neuroendocrinol.* 26 724–738. 10.1111/jne.12182 25059307

[B17] Halperin J. M. Healey D. M. (2011). The influences of environmental enrichment, cognitive enhancement, and physical exercise on brain development: can we alter the developmental trajectory of ADHD? *Neurosci. Biobehav. Rev.* 35 621–634. 10.1016/j.neubiorev.2010.07.006 20691725 PMC3008505

[B18] Leggio M. G. Mandolesi L. Federico F. Spirito F. Ricci B. Gelfo F. (2005). Environmental enrichment promotes improved spatial abilities and enhanced dendritic growth in the rat. *Behav. Brain Res.* 163 78–90. 10.1016/j.bbr.2005.04.009 15913801

[B19] Lehmann J. Pryce C. R. Feldon J. (2000). Lack of effect of an early stressful life event on sensorimotor gating in adult rats. *Schizophr. Res.* 41 365–371. 10.1016/S0920-9964(99)00080-8 10708346

[B20] Levine S. (2005). Developmental determinants of sensitivity and resistance to stress. *Psychoneuroendocrinology* 30 939–946. 10.1016/j.psyneuen.2005.03.013 15958281

[B21] Lovic V. Keen D. Fletcher P. J. Fleming A. S. (2011). Early-life maternal separation and social isolation produce an increase in impulsive action but not impulsive choice. *Behav. Neurosci.* 125 481–491. 10.1037/a0024367 21688886

[B22] Marco E. M. Llorente R. Lopez-Gallardo M. Mela V. Llorente-Berzal A. Prada C. (2015). The maternal deprivation animal model revisited. *Neurosci. Biobehav. Rev.* 51 151–163. 10.1016/j.neubiorev.2015.01.015 25616179

[B23] Meaney M. J. (2010). Epigenetics and the biological definition of gene x environment interactions. *Child Dev.* 81 41–79. 10.1111/j.1467-8624.2009.01381.x 20331654

[B24] Moffitt T. E. Arseneault L. Belsky D. Dickson N. Hancox R. J. Harrington H. (2011). A gradient of childhood self-control predicts health, wealth, and public safety. *Proc. Natl. Acad. Sci. U.S.A.* 108 2693–2698. 10.1073/pnas.1010076108 21262822 PMC3041102

[B25] Nederhof E. Schmidt M. V. (2012). Mismatch or cumulative stress: toward an integrated hypothesis of programming effects. *Physiol. Behav.* 106 691–700. 10.1016/j.physbeh.2011.12.008 22210393

[B26] O’Donnell K. A. Gaudreau H. Colalillo S. Steiner M. Atkinson L. Moss E. (2014). The maternal adversity, vulnerability and neurodevelopment project: theory and methodology. *Can. J. Psychiatry* 59 497–508. 25565695 10.1177/070674371405900906 PMC4168812

[B27] Oomen C. A. Soeters H. Audureau N. Vermunt L. van Hasselt F. N. Manders E. M. (2010). Severe early life stress hampers spatial learning and neurogenesis, but improves hippocampal synaptic plasticity and emotional learning under high-stress conditions in adulthood. *J. Neurosci.* 30 6635–6645. 10.1523/jneurosci.0247-10.2010 20463226 PMC6632559

[B28] Perry J. L. Carroll M. E. (2008). The role of impulsive behavior in drug abuse. *Psychopharmacology (Berl.)* 200 1–26. 10.1007/s00213-008-1173-0 18600315

[B29] Perry J. L. Stairs D. J. Bardo M. T. (2008). Impulsive choice and environmental enrichment: effects of d-amphetamine and methylphenidate. *Behav. Brain Res.* 193 48–54. 10.1016/j.bbr.2008.04.019 18534693 PMC2681296

[B30] Petersen S. E. Posner M. I. (2012). The attention system of the human brain: 20 years after. *Annu. Rev. Neurosci.* 35 73–89. 10.1146/annurev-neuro-062111-150525 22524787 PMC3413263

[B31] Pryce C. R. Feldon J. (2003). Long-term neurobehavioural impact of the postnatal environment in rats: manipulations, effects and mediating mechanisms. *Neurosci. Biobehav. Rev.* 27 57–71. 10.1016/S0149-7634(03)00009-5 12732223

[B32] Robbins T. W. (2002). The 5-choice serial reaction time task: behavioural pharmacology and functional neurochemistry. *Psychopharmacology (Berl.)* 163 362–380. 10.1007/s00213-002-1154-7 12373437

[B33] Robinson T. E. Yager L. M. Cogan E. S. Saunders B. T. (2014). On the motivational properties of reward cues: Individual differences. *Neuropharmacology* 76(Pt B), 450–459. 10.1016/j.neuropharm.2013.05.040 23748094 PMC3796005

[B34] Rosenzweig M. R. Bennett E. L. (1996). Psychobiology of plasticity: effects of training and experience on brain and behavior. *Behav. Brain Res.* 78 57–65. 10.1016/0166-4328(95)00216-2 8793038

[B35] Rots N. Y. de Jong J. Workel J. O. Levine S. Cools A. R. De Kloet E. R. (1996). Neonatal maternally deprived rats have as adults elevated basal pituitary-adrenal activity and enhanced susceptibility to apomorphine. *J. Neuroendocrinol.* 8 501–506. 10.1046/j.1365-2826.1996.04843.x 8843018

[B36] Solinas M. Thiriet N. Chauvet C. Jaber M. (2010). Prevention and treatment of drug addiction by environmental enrichment. *Prog. Neurobiol.* 92 572–592. 10.1016/j.pneurobio.2010.08.002 20713127

[B37] Spivey J. M. Shumake J. Colorado R. A. Conejo-Jimenez N. Gonzalez-Pardo H. Gonzalez-Lima F. (2009). Adolescent female rats are more resistant than males to the effects of early stress on prefrontal cortex and impulsive behavior. *Dev. Psychobiol.* 51 277–288. 10.1002/dev.20362 19125421 PMC2754836

[B38] Stairs D. J. Bardo M. T. (2009). Neurobehavioral effects of environmental enrichment and drug abuse vulnerability. *Pharmacol. Biochem. Behav.* 92 377–382. 10.1016/j.pbb.2009.01.016 19463254 PMC2687322

[B39] Sullivan R. M. Brake W. G. (2003). What the rodent prefrontal cortex can teach us about attention-deficit/hyperactivity disorder: the critical role of early developmental events on prefrontal function. *Behav. Brain Res.* 146 43–55. 10.1016/j.bbr.2003.09.015 14643458

[B40] Tabachnick B. G. Fidell L. S. (2006). *Using Multivariate Statistics*, 5th Edn. Boston, MA: Allyn & Bacon, Inc.

[B41] Tunstall B. J. Kearns D. N. (2015). Sign-tracking predicts increased choice of cocaine over food in rats. *Behav. Brain Res.* 281 222–228. 10.1016/j.bbr.2014.12.034 25541036 PMC4305489

[B42] Van den Buuse M. Garner B. Koch M. (2003). Neurodevelopmental animal models of schizophrenia: effects on prepulse inhibition. *Curr. Mol. Med.* 3 459–471. 10.2174/1566524033479627 12942999

[B43] van Oers H. J. de Kloet E. R. Levine S. (1997). Persistent, but paradoxical, effects on HPA regulation of infants maternally deprived at different ages. *Stress* 1 249–262. 9787249 10.3109/10253899709013745

[B44] van Praag H. Kempermann G. Gage F. H. (2000). Neural consequences of environmental enrichment. *Nat. Rev. Neurosci.* 1 191–198. 10.1038/35044558 11257907

[B45] Winstanley C. A. Eagle D. M. Robbins T. W. (2006). Behavioral models of impulsivity in relation to ADHD: translation between clinical and preclinical studies. *Clin. Psychol. Rev.* 26 379–395. 10.1016/j.cpr.2006.01.001 16504359 PMC1892795

[B46] Wurbel H. (2001). Ideal homes? Housing effects on rodent brain and behaviour. *Trends Neurosci.* 24 207–211. 10.1016/S0166-2236(00)01718-5 11250003

[B47] Zhang Y. Crofton E. J. Li D. Lobo M. K. Fan X. Nestler E. J. (2014). Overexpression of DeltaFosB in nucleus accumbens mimics the protective addiction phenotype, but not the protective depression phenotype of environmental enrichment. *Front. Behav. Neurosci.* 8:297 10.3389/fnbeh.2014.00297 PMC4148937 25221490

[B48] Zimmermann A. Stauffacher M. Langhans W. Wurbel H. (2001). Enrichment-dependent differences in novelty exploration in rats can be explained by habituation. *Behav. Brain Res.* 121 11–20. 10.1016/S0166-4328(00)00377-6 11275280

